# Dimeric (2-cyano­phenolato-κ*O*){2,2′-[ethyl­enebis(nitrilo­methyl­idyne)]diphenolato-κ^4^ *O*,*N*,*N*′,*O*′}manganese(III) monohydrate

**DOI:** 10.1107/S1600536811026584

**Published:** 2011-07-09

**Authors:** Youcef Zidane, Ali Ourari, Hénia Mousser, Abdelhamid Mousser

**Affiliations:** a Laboratoire d’Électrochimie, d’Ingénierie Moléculaire et de Catalyse Redox (LEIMCR), Faculté des Sciences de l’Ingénieur, Université Farhat Abbas de Sétif 19000, Algeria; b Département de Chimie Industrielle, Faculté des Sciences de l’Ingénieur, Université Mentouri Constantine, Campus Chaab Erssas, Constantine, Algeria; c Département de Chimie, Faculté des Sciences Exactes, Université Mentouri Constantine, Route de Ain El Bey, Constantine, Algeria

## Abstract

The molecules of the title compound, [Mn(C_7_H_4_NO)(C_16_H_14_N_2_O_2_)]·H_2_O, form dimers in the solid state across a crystallographic inversion center. The bridging Mn_2_O_2_ group is built of phen­oxy groups, and is asymmetric, with an Mn—O distances of 1.9002 (13) and 2.6236 (14) Å. A substantial cavity between the two Mn atoms [Mn⋯Mn = 3.5082 (4) Å] is produced by the formation of the dimer. In the crystal, an extended network of O—H⋯O hydrogen-bonding inter­actions stabil­izes the structure.

## Related literature

For related structures, see: Mirkhani *et al.* (2006 ▶); Oyaizu *et al.* (2000 ▶); Zhang *et al.* (2009 ▶). For applications of Mn^II^ complexes in catalysis, see: Ourari *et al.* (2006 ▶, 2008 ▶); Srinivasan *et al.* (1986 ▶); Salomao *et al.* (2007 ▶); Moutet & Ourari (1997 ▶). For the synthesis, see: Trivedi *et al.* (1992 ▶).
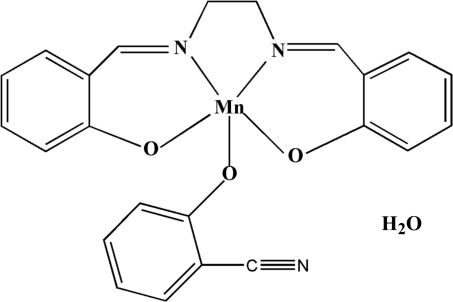


## Experimental

### 

#### Crystal data

[Mn(C_7_H_4_NO)(C_16_H_12_N_2_O_2_)]·H_2_O
*M* *_r_* = 457.36
Monoclinic, 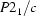

*a* = 12.8693 (3) Å
*b* = 14.2487 (3) Å
*c* = 11.6357 (3) Å
β = 104.297 (1)°
*V* = 2067.57 (8) Å^3^
*Z* = 4
Mo *K*α radiation
μ = 0.67 mm^−1^
*T* = 293 K
0.08 × 0.06 × 0.04 mm

#### Data collection

Bruker APEXII CCD area-detector diffractometer
8907 measured reflections
4732 independent reflections
3574 reflections with *I* > 2σ(*I*)
*R*_int_ = 0.024

#### Refinement

*R*[*F* ^2^ > 2σ(*F* ^2^)] = 0.038
*wR*(*F* ^2^) = 0.103
*S* = 1.05
4601 reflections
288 parameters
H atoms treated by a mixture of independent and constrained refinement
Δρ_max_ = 0.36 e Å^−3^
Δρ_min_ = −0.42 e Å^−3^

### 

Data collection: *APEX2* (Bruker, 2006 ▶); cell refinement: *SAINT* (Bruker, 2006 ▶); data reduction: *SAINT*; program(s) used to solve structure: *SHELXS97* (Sheldrick, 2008 ▶); program(s) used to refine structure: *SHELXL97* (Sheldrick, 2008 ▶); molecular graphics: *ORTEPIII* (Burnett & Johnson, 1996 ▶); software used to prepare material for publication: *SHELXL97*.

## Supplementary Material

Crystal structure: contains datablock(s) I, global. DOI: 10.1107/S1600536811026584/om2428sup1.cif

Structure factors: contains datablock(s) I. DOI: 10.1107/S1600536811026584/om2428Isup2.hkl

Additional supplementary materials:  crystallographic information; 3D view; checkCIF report

## Figures and Tables

**Table 1:** Hydrogen-bond geometry (Å, °)

*D*—H⋯*A*	*D*—H	H⋯*A*	*D*⋯*A*	*D*—H⋯*A*
O1*W*—H1*W*⋯O2	0.86 (4)	2.24 (5)	2.959 (3)	141 (4)
O1*W*—H2*W*⋯O3^i^	0.96 (4)	1.91 (4)	2.840 (3)	163 (4)

Symmetry code: (i) 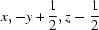.

**Fig. 1.**
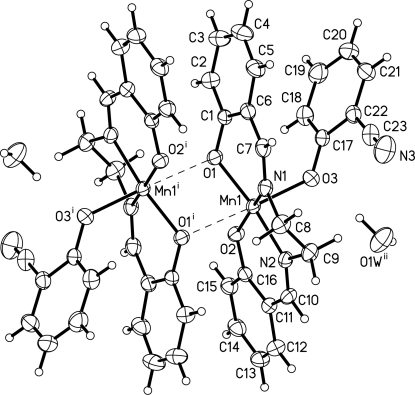
The molecular structure of the title compound with atom labels and 35% probability displacement ellipsoids for non-H atoms. Symmetry codes: i = 1-x, 1-y, 2-z; ii = 0.5+x, 0.5-y, 1+z.

**Fig. 2.**
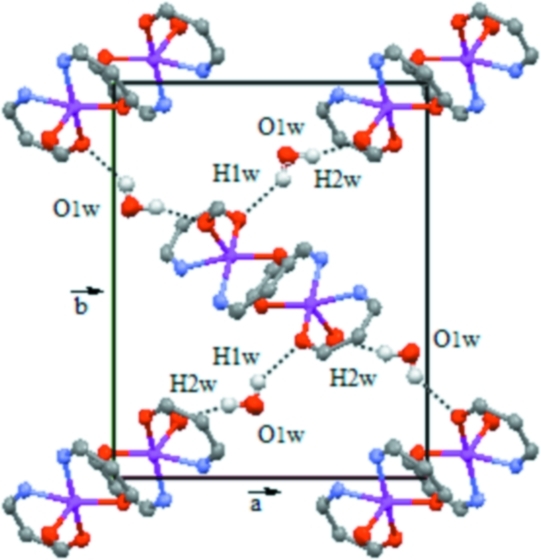
The packing of the title compound, viewed down the c axis, showing one layer of molecules connected by O—H···O hydrogen bonds (dashed lines). The phenyl rings and H atoms out of the clusters have been omitted for clarity.

## supplementary crystallographic information

### Comment

Tetradentate Schiff base complexes of transition metals were involved in a large research activity during the last ten years. The complexes of manganese(III) are currently used in catalysis (Ourari *et al.*, 2006; Ourari *et al.*, 2008; Srinivasan *et al.*, 1986; Salomao *et al.*, 2007) and in electrocatalysis reactions (Moutet, *et al.*, 1997). In the present paper, we describe the synthesis and structural study of the dimeric 2-cyanophenoxy{2,2-[ethylenebis-(nitrilomethylidyne)]diphenolato}-manganese(III) monohydrate. The metal fragment in this complex is similar to those described by Mirkhani *et al.*, 2006; Oyaizu *et al.*, 2000; Zhang *et  al.*, 2009), where the manganese(III) is chelated by an imidazole moiety, arylcarboxylate group and azide ion, respectively. In the title compound, the manganese(III) atom is chelated by the Schiff base ligand *via* two N and two O atoms, and is additionally coordinated by a cyanophenoxy group (Fig. 1), forming a distorted MnN_2_O_3_ square pyramidal arrangement (Table 1). The Schiff base lies in the equatorial plane, and the cyanophenoxy group is in the axial coordination site. The manganese(III) atom is diplaced out of the salen N_2_O_2_ plane by 0.18Å towards the cyanophenoxy group and confirms that the coordination geometry around the manganese is susceptible to the nature of the axial ligand as described by Oyaizu *et al.*, 2000. In the crystal packing, 2-cyanophenoxy{2,2-[ethylenebis-(nitrilomethylidyne)]diphenolato} -manganese(III) monohydrate forms a solid-state dimer in which the Mn(salen) moiety is linked to its neighbor by two shared cyanophenoxy oxygen atoms. So each manganese(III) achieves a distorted octahedral geometry (Table 1). The Mn—Mn distance is 3.5082 (4)Å. An extended network of O—H···O (Table 2) hydrogen-bonding interactions stabilizes the solid state (Fig. 2). Each dimer is linked to four neighbor dimers *via* hydrogen water molecule bonds to give layers parallel to (001) plane.

### Experimental

Reagent grade 2-hydroxybenzaldehyde (Aldrich), 1,2-diaminoethane (Fluka) and manganese(II) acetate tetrahydrate (Fluka) were obtained commercially. All the solvents used were of reagent grade. The symmetrical Schiff base was prepared in one step synthesis according to the method described by Trivedi *et al.*, 1992. The ligand was obtained by refluxing a mixture of 2 mmol of 2-hydroxybenzaldehyde and 1 mmol of 1,2-diaminoethane in 25 ml of ethanol for 30 min. A methanolic solution (15 ml) of Mn(Ac)_2_, 4H_2_O (1 mmol) was added dropwise to the resulting yellow solution containing the ligand.  The reaction mixture was stirred continuously for 24 hours under air atmosphere and the mixture was filtered. The filtrate was concentrated to ca. 20 ml under reduced pressure, kept for several weeks at ambient temperature. The brown crystals were collected by filtration and were washed thoroughly with a minimum amount of methanol and dried in air. Yield: (78%).

### Refinement

H atoms were positined geometrically, using a riding model with C—H = 0.96 Å (*U*_iso_(H) = 1.5) (including free rotation about C—C and C—N bond) for methyl groups and with C—H = 0.93 and 0.97 Å (1.2 for aromatic and methylene groups) times *U*_eq_(C). Hydrogen atoms bonded to the water oxygen were freely refined.

The difference between 4732 independent reflections and 4601 reflections used in the refinement is 131 reflections. These are omitted, in refinement, as bad reflections.

### Crystal data

**Table:**

[Mn(C_7_H_4_NO)(C_16_H_14_N_2_O_2_)]·H_2_O	*F*(000) = 944
*M**_r_* = 457.36	*D*_x_ = 1.469 Mg m^−^^3^
Monoclinic, *P*2_1_/*c*	Mo *K*α radiation, λ = 0.71073 Å
Hall symbol: -P 2ybc	Cell parameters from 3574 reflections
*a* = 12.8693 (3) Å	θ = 1.0–27.5°
*b* = 14.2487 (3) Å	µ = 0.67 mm^−^^1^
*c* = 11.6357 (3) Å	*T* = 293 K
β = 104.297 (1)°	Prism, brown
*V* = 2067.57 (8) Å^3^	0.08 × 0.06 × 0.04 mm
*Z* = 4	

### Data collection

**Table:**

Bruker APEXII CCD area-detector diffractometer	3574 reflections with *I* > 2σ(*I*)
Radiation source: fine-focus sealed tube	*R*_int_ = 0.024
graphite	θ_max_ = 27.5°, θ_min_ = 3.1°
Detector resolution: 8.192 pixels mm^-1^	*h* = −16→16
ω scans	*k* = −18→18
8907 measured reflections	*l* = −15→15
4732 independent reflections	

### Refinement

**Table:**

Refinement on *F*^2^	Primary atom site location: structure-invariant direct methods
Least-squares matrix: full	Secondary atom site location: difference Fourier map
*R*[*F*^2^ > 2σ(*F*^2^)] = 0.038	Hydrogen site location: inferred from neighbouring sites
*wR*(*F*^2^) = 0.103	H atoms treated by a mixture of independent and constrained refinement
*S* = 1.05	*w* = 1/[σ^2^(*F*_o_^2^) + (0.0533*P*)^2^ + 0.4975*P*] where *P* = (*F*_o_^2^ + 2*F*_c_^2^)/3
4601 reflections	(Δ/σ)_max_ = 0.003
288 parameters	Δρ_max_ = 0.36 e Å^−^^3^
0 restraints	Δρ_min_ = −0.42 e Å^−^^3^

### Special details

**Table:**

Geometry. All s.u.'s (except the s.u. in the dihedral angle between two l.s. planes) are estimated using the full covariance matrix. The cell s.u.'s are taken into account individually in the estimation of s.u.'s in distances, angles and torsion angles; correlations between s.u.'s in cell parameters are only used when they are defined by crystal symmetry. An approximate (isotropic) treatment of cell s.u.'s is used for estimating s.u.'s involving l.s. planes.
Refinement. Refinement of *F*^2^ against ALL reflections. The weighted *R*-factor *wR* and goodness of fit *S* are based on *F*^2^, conventional *R*-factors *R* are based on *F*, with *F* set to zero for negative *F*^2^. The threshold expression of *F*^2^ > σ(*F*^2^) is used only for calculating *R*-factors(gt) *etc*. and is not relevant to the choice of reflections for refinement. *R*-factors based on *F*^2^ are statistically about twice as large as those based on *F*, and *R*- factors based on ALL data will be even larger.

### Fractional atomic coordinates and isotropic or equivalent isotropic displacement parameters (Å^2^)

**Table:**

	*x*	*y*	*z*	*U*_iso_*/*U*_eq_	
Mn1	0.99092 (2)	0.43893 (2)	0.62805 (2)	0.02983 (10)	
O1	0.90494 (10)	0.43871 (10)	0.46974 (11)	0.0357 (3)	
O2	1.08666 (10)	0.34558 (9)	0.60129 (11)	0.0350 (3)	
O3	0.89791 (11)	0.35388 (11)	0.70612 (13)	0.0448 (4)	
N1	0.91679 (14)	0.55496 (11)	0.66080 (14)	0.0354 (4)	
N2	1.08619 (13)	0.46912 (11)	0.78489 (13)	0.0339 (3)	
N3	0.7505 (2)	0.48210 (18)	0.8743 (2)	0.0767 (7)	
C1	0.79945 (15)	0.45831 (14)	0.44352 (17)	0.0337 (4)	
C2	0.73190 (17)	0.41319 (16)	0.34833 (19)	0.0435 (5)	
H2	0.7596	0.3694	0.3046	0.052*	
C3	0.62331 (18)	0.43313 (17)	0.3182 (2)	0.0525 (6)	
H3	0.5786	0.4019	0.2547	0.063*	
C4	0.58015 (18)	0.49842 (19)	0.3805 (2)	0.0568 (6)	
H4	0.5068	0.5102	0.3599	0.068*	
C5	0.64539 (18)	0.54566 (17)	0.4724 (2)	0.0483 (5)	
H5	0.6166	0.5916	0.5122	0.058*	
C6	0.75579 (16)	0.52575 (14)	0.50774 (18)	0.0373 (4)	
C7	0.82058 (17)	0.57678 (15)	0.60655 (18)	0.0397 (5)	
H7	0.7904	0.6294	0.6329	0.048*	
C8	0.97976 (19)	0.60968 (15)	0.76127 (19)	0.0445 (5)	
H8A	0.9330	0.6482	0.7954	0.053*	
H8B	1.0302	0.6502	0.7356	0.053*	
C9	1.03842 (19)	0.53916 (16)	0.85072 (18)	0.0426 (5)	
H9A	1.0941	0.5700	0.9103	0.051*	
H9B	0.9891	0.5089	0.8900	0.051*	
C10	1.17722 (17)	0.43171 (15)	0.83253 (17)	0.0387 (4)	
H10	1.2138	0.4538	0.9067	0.046*	
C11	1.22743 (15)	0.35839 (14)	0.78078 (17)	0.0362 (4)	
C12	1.32795 (17)	0.32527 (17)	0.84575 (19)	0.0465 (5)	
H12	1.3599	0.3530	0.9182	0.056*	
C13	1.38001 (18)	0.25341 (17)	0.8053 (2)	0.0488 (5)	
H13	1.4465	0.2327	0.8492	0.059*	
C14	1.33099 (18)	0.21203 (16)	0.6970 (2)	0.0460 (5)	
H14	1.3648	0.1623	0.6692	0.055*	
C15	1.23384 (17)	0.24326 (14)	0.63047 (19)	0.0405 (5)	
H15	1.2033	0.2148	0.5581	0.049*	
C16	1.17987 (15)	0.31754 (13)	0.66996 (16)	0.0328 (4)	
C17	0.80118 (16)	0.31955 (14)	0.66939 (17)	0.0365 (4)	
C18	0.7748 (2)	0.25034 (16)	0.5805 (2)	0.0474 (5)	
H18	0.8274	0.2283	0.5451	0.057*	
C19	0.6724 (2)	0.21485 (19)	0.5453 (2)	0.0584 (6)	
H19	0.6570	0.1695	0.4860	0.070*	
C20	0.5921 (2)	0.2451 (2)	0.5959 (2)	0.0607 (7)	
H20	0.5235	0.2200	0.5715	0.073*	
C21	0.61415 (18)	0.31221 (18)	0.6821 (2)	0.0517 (6)	
H21	0.5603	0.3329	0.7166	0.062*	
C22	0.71671 (17)	0.34976 (15)	0.71865 (18)	0.0404 (5)	
C23	0.7380 (2)	0.42338 (17)	0.8061 (2)	0.0514 (6)	
O1W	0.9967 (2)	0.18475 (15)	0.44789 (18)	0.0744 (6)	
H2W	0.962 (3)	0.185 (3)	0.366 (3)	0.110 (13)*	
H1W	1.004 (4)	0.244 (3)	0.461 (4)	0.125 (16)*	

### Atomic displacement parameters (Å^2^)

**Table:**

	*U*^11^	*U*^22^	*U*^33^	*U*^12^	*U*^13^	*U*^23^
Mn1	0.03033 (16)	0.03349 (17)	0.02616 (15)	0.00335 (11)	0.00787 (11)	−0.00182 (11)
O1	0.0314 (7)	0.0465 (8)	0.0293 (6)	0.0064 (6)	0.0079 (5)	−0.0036 (6)
O2	0.0332 (7)	0.0383 (7)	0.0326 (7)	0.0049 (6)	0.0061 (5)	−0.0033 (5)
O3	0.0369 (7)	0.0539 (9)	0.0434 (8)	−0.0077 (7)	0.0098 (6)	0.0072 (7)
N1	0.0408 (9)	0.0361 (9)	0.0321 (8)	0.0045 (7)	0.0142 (7)	−0.0014 (7)
N2	0.0382 (9)	0.0366 (8)	0.0276 (8)	−0.0009 (7)	0.0095 (6)	−0.0017 (6)
N3	0.110 (2)	0.0640 (15)	0.0607 (14)	0.0043 (14)	0.0301 (14)	−0.0113 (12)
C1	0.0298 (9)	0.0394 (10)	0.0326 (9)	0.0020 (8)	0.0091 (7)	0.0074 (8)
C2	0.0399 (11)	0.0444 (11)	0.0442 (12)	0.0010 (9)	0.0069 (9)	−0.0002 (9)
C3	0.0380 (12)	0.0519 (14)	0.0613 (14)	−0.0043 (10)	0.0004 (10)	0.0004 (11)
C4	0.0311 (11)	0.0618 (15)	0.0754 (17)	0.0032 (11)	0.0092 (11)	0.0041 (13)
C5	0.0368 (11)	0.0511 (13)	0.0599 (14)	0.0107 (9)	0.0176 (10)	0.0056 (11)
C6	0.0353 (10)	0.0387 (10)	0.0403 (10)	0.0041 (8)	0.0136 (8)	0.0076 (8)
C7	0.0429 (11)	0.0402 (11)	0.0406 (11)	0.0090 (9)	0.0194 (9)	0.0016 (9)
C8	0.0521 (13)	0.0398 (11)	0.0413 (11)	0.0067 (10)	0.0107 (9)	−0.0108 (9)
C9	0.0515 (12)	0.0445 (11)	0.0325 (10)	0.0013 (9)	0.0120 (9)	−0.0093 (8)
C10	0.0403 (11)	0.0452 (11)	0.0273 (9)	−0.0031 (9)	0.0022 (8)	−0.0021 (8)
C11	0.0341 (10)	0.0403 (10)	0.0336 (10)	0.0007 (8)	0.0074 (8)	0.0046 (8)
C12	0.0400 (11)	0.0555 (13)	0.0399 (11)	0.0009 (10)	0.0022 (9)	0.0040 (10)
C13	0.0331 (11)	0.0582 (14)	0.0526 (13)	0.0081 (10)	0.0058 (9)	0.0122 (11)
C14	0.0373 (11)	0.0460 (12)	0.0578 (13)	0.0090 (9)	0.0180 (10)	0.0080 (10)
C15	0.0379 (11)	0.0413 (11)	0.0435 (11)	0.0037 (9)	0.0122 (9)	0.0005 (9)
C16	0.0299 (9)	0.0347 (10)	0.0351 (9)	0.0001 (7)	0.0106 (7)	0.0053 (8)
C17	0.0385 (10)	0.0373 (10)	0.0343 (10)	−0.0023 (8)	0.0099 (8)	0.0085 (8)
C18	0.0549 (14)	0.0459 (12)	0.0447 (12)	−0.0022 (10)	0.0188 (10)	−0.0012 (10)
C19	0.0704 (17)	0.0579 (15)	0.0442 (13)	−0.0176 (13)	0.0089 (12)	−0.0077 (11)
C20	0.0450 (14)	0.0773 (18)	0.0549 (14)	−0.0177 (12)	0.0029 (11)	0.0073 (13)
C21	0.0392 (12)	0.0618 (15)	0.0560 (14)	0.0011 (11)	0.0150 (10)	0.0109 (11)
C22	0.0429 (11)	0.0412 (11)	0.0393 (10)	0.0008 (9)	0.0144 (9)	0.0056 (9)
C23	0.0620 (15)	0.0505 (13)	0.0463 (13)	0.0017 (11)	0.0223 (11)	0.0034 (11)
O1W	0.1058 (17)	0.0567 (12)	0.0507 (11)	0.0004 (11)	0.0002 (11)	−0.0084 (9)

### Geometric parameters (Å, °)

**Table:**

Mn1—O2	1.8903 (13)	C8—H8B	0.9700
Mn1—O1	1.9002 (13)	C9—H9A	0.9700
Mn1—N2	1.9782 (16)	C9—H9B	0.9700
Mn1—N1	1.9921 (16)	C10—C11	1.436 (3)
Mn1—O3	2.0649 (14)	C10—H10	0.9300
Mn1—O1^i^	2.6236 (14)	C11—C12	1.409 (3)
O1—C1	1.345 (2)	C11—C16	1.409 (3)
O2—C16	1.328 (2)	C12—C13	1.369 (3)
O3—C17	1.307 (2)	C12—H12	0.9300
N1—C7	1.282 (3)	C13—C14	1.394 (3)
N1—C8	1.471 (3)	C13—H13	0.9300
N2—C10	1.282 (3)	C14—C15	1.372 (3)
N2—C9	1.481 (3)	C14—H14	0.9300
N3—C23	1.137 (3)	C15—C16	1.404 (3)
C1—C2	1.386 (3)	C15—H15	0.9300
C1—C6	1.416 (3)	C17—C18	1.408 (3)
C2—C3	1.384 (3)	C17—C22	1.415 (3)
C2—H2	0.9300	C18—C19	1.377 (4)
C3—C4	1.378 (4)	C18—H18	0.9300
C3—H3	0.9300	C19—C20	1.379 (4)
C4—C5	1.363 (4)	C19—H19	0.9300
C4—H4	0.9300	C20—C21	1.364 (4)
C5—C6	1.407 (3)	C20—H20	0.9300
C5—H5	0.9300	C21—C22	1.390 (3)
C6—C7	1.440 (3)	C21—H21	0.9300
C7—H7	0.9300	C22—C23	1.440 (3)
C8—C9	1.508 (3)	O1W—H2W	0.95 (4)
C8—H8A	0.9700	O1W—H1W	0.85 (4)
			
O2—Mn1—O1	94.96 (6)	N2—C9—H9A	110.3
O2—Mn1—N2	91.39 (6)	C8—C9—H9A	110.3
O1—Mn1—N2	167.03 (7)	N2—C9—H9B	110.3
O2—Mn1—N1	167.92 (7)	C8—C9—H9B	110.3
O1—Mn1—N1	89.72 (6)	H9A—C9—H9B	108.6
N2—Mn1—N1	81.95 (7)	N2—C10—C11	125.37 (18)
O2—Mn1—O3	97.57 (6)	N2—C10—H10	117.3
O1—Mn1—O3	99.41 (6)	C11—C10—H10	117.3
N2—Mn1—O3	90.94 (6)	C12—C11—C16	119.10 (19)
N1—Mn1—O3	92.63 (6)	C12—C11—C10	117.85 (18)
C1—O1—Mn1	122.21 (11)	C16—C11—C10	123.04 (18)
C16—O2—Mn1	129.88 (12)	C13—C12—C11	121.9 (2)
C17—O3—Mn1	133.20 (13)	C13—C12—H12	119.0
C7—N1—C8	122.58 (17)	C11—C12—H12	119.0
C7—N1—Mn1	123.87 (14)	C12—C13—C14	118.5 (2)
C8—N1—Mn1	113.34 (13)	C12—C13—H13	120.8
C10—N2—C9	120.55 (16)	C14—C13—H13	120.8
C10—N2—Mn1	126.90 (14)	C15—C14—C13	121.3 (2)
C9—N2—Mn1	112.45 (13)	C15—C14—H14	119.4
O1—C1—C2	118.93 (18)	C13—C14—H14	119.4
O1—C1—C6	122.07 (17)	C14—C15—C16	121.0 (2)
C2—C1—C6	118.97 (18)	C14—C15—H15	119.5
C3—C2—C1	120.1 (2)	C16—C15—H15	119.5
C3—C2—H2	119.9	O2—C16—C15	118.44 (18)
C1—C2—H2	119.9	O2—C16—C11	123.35 (17)
C4—C3—C2	121.2 (2)	C15—C16—C11	118.21 (18)
C4—C3—H3	119.4	O3—C17—C18	122.64 (19)
C2—C3—H3	119.4	O3—C17—C22	121.23 (19)
C5—C4—C3	119.7 (2)	C18—C17—C22	116.13 (19)
C5—C4—H4	120.1	C19—C18—C17	121.0 (2)
C3—C4—H4	120.1	C19—C18—H18	119.5
C4—C5—C6	120.8 (2)	C17—C18—H18	119.5
C4—C5—H5	119.6	C18—C19—C20	121.4 (2)
C6—C5—H5	119.6	C18—C19—H19	119.3
C5—C6—C1	119.1 (2)	C20—C19—H19	119.3
C5—C6—C7	118.39 (19)	C21—C20—C19	119.5 (2)
C1—C6—C7	122.49 (18)	C21—C20—H20	120.3
N1—C7—C6	124.71 (19)	C19—C20—H20	120.3
N1—C7—H7	117.6	C20—C21—C22	120.3 (2)
C6—C7—H7	117.6	C20—C21—H21	119.9
N1—C8—C9	106.19 (17)	C22—C21—H21	119.9
N1—C8—H8A	110.5	C21—C22—C17	121.7 (2)
C9—C8—H8A	110.5	C21—C22—C23	119.8 (2)
N1—C8—H8B	110.5	C17—C22—C23	118.5 (2)
C9—C8—H8B	110.5	N3—C23—C22	177.3 (3)
H8A—C8—H8B	108.7	H2W—O1W—H1W	100 (4)
N2—C9—C8	107.04 (16)		
			
O2—Mn1—O1—C1	149.32 (14)	C8—N1—C7—C6	179.83 (19)
N2—Mn1—O1—C1	−91.7 (3)	Mn1—N1—C7—C6	−5.8 (3)
N1—Mn1—O1—C1	−41.83 (15)	C5—C6—C7—N1	167.2 (2)
O3—Mn1—O1—C1	50.79 (15)	C1—C6—C7—N1	−14.3 (3)
O1—Mn1—O2—C16	169.06 (15)	C7—N1—C8—C9	138.9 (2)
N2—Mn1—O2—C16	0.38 (16)	Mn1—N1—C8—C9	−36.0 (2)
N1—Mn1—O2—C16	56.6 (4)	C10—N2—C9—C8	145.0 (2)
O3—Mn1—O2—C16	−90.75 (16)	Mn1—N2—C9—C8	−38.2 (2)
O2—Mn1—O3—C17	−106.03 (19)	N1—C8—C9—N2	46.5 (2)
O1—Mn1—O3—C17	−9.7 (2)	C9—N2—C10—C11	175.19 (19)
N2—Mn1—O3—C17	162.44 (19)	Mn1—N2—C10—C11	−1.0 (3)
N1—Mn1—O3—C17	80.46 (19)	N2—C10—C11—C12	−179.9 (2)
O2—Mn1—N1—C7	140.7 (3)	N2—C10—C11—C16	−1.1 (3)
O1—Mn1—N1—C7	27.74 (16)	C16—C11—C12—C13	−1.2 (3)
N2—Mn1—N1—C7	−162.23 (17)	C10—C11—C12—C13	177.7 (2)
O3—Mn1—N1—C7	−71.66 (17)	C11—C12—C13—C14	−0.4 (3)
O2—Mn1—N1—C8	−44.5 (4)	C12—C13—C14—C15	1.3 (3)
O1—Mn1—N1—C8	−157.44 (14)	C13—C14—C15—C16	−0.6 (3)
N2—Mn1—N1—C8	12.58 (14)	Mn1—O2—C16—C15	177.54 (13)
O3—Mn1—N1—C8	103.15 (14)	Mn1—O2—C16—C11	−2.3 (3)
O2—Mn1—N2—C10	1.26 (17)	C14—C15—C16—O2	179.23 (18)
O1—Mn1—N2—C10	−118.1 (3)	C14—C15—C16—C11	−0.9 (3)
N1—Mn1—N2—C10	−168.63 (18)	C12—C11—C16—O2	−178.37 (18)
O3—Mn1—N2—C10	98.85 (18)	C10—C11—C16—O2	2.8 (3)
O2—Mn1—N2—C9	−175.21 (14)	C12—C11—C16—C15	1.8 (3)
O1—Mn1—N2—C9	65.4 (3)	C10—C11—C16—C15	−177.01 (18)
N1—Mn1—N2—C9	14.90 (13)	Mn1—O3—C17—C18	69.0 (3)
O3—Mn1—N2—C9	−77.62 (14)	Mn1—O3—C17—C22	−111.8 (2)
Mn1—O1—C1—C2	−146.56 (15)	O3—C17—C18—C19	179.1 (2)
Mn1—O1—C1—C6	35.5 (2)	C22—C17—C18—C19	−0.2 (3)
O1—C1—C2—C3	−179.0 (2)	C17—C18—C19—C20	−0.4 (4)
C6—C1—C2—C3	−1.0 (3)	C18—C19—C20—C21	0.5 (4)
C1—C2—C3—C4	0.8 (4)	C19—C20—C21—C22	0.0 (4)
C2—C3—C4—C5	1.1 (4)	C20—C21—C22—C17	−0.7 (4)
C3—C4—C5—C6	−2.7 (4)	C20—C21—C22—C23	177.4 (2)
C4—C5—C6—C1	2.4 (3)	O3—C17—C22—C21	−178.5 (2)
C4—C5—C6—C7	−179.1 (2)	C18—C17—C22—C21	0.8 (3)
O1—C1—C6—C5	177.34 (18)	O3—C17—C22—C23	3.4 (3)
C2—C1—C6—C5	−0.6 (3)	C18—C17—C22—C23	−177.28 (19)
O1—C1—C6—C7	−1.1 (3)	C21—C22—C23—N3	−16 (6)
C2—C1—C6—C7	−179.04 (19)	C17—C22—C23—N3	162 (6)

Symmetry codes: (i) −*x*+1, −*y*+1, −*z*+2.

### Hydrogen-bond geometry (Å, °)

**Table:**

*D*—H···*A*	*D*—H	H···*A*	*D*···*A*	*D*—H···*A*
O1W—H1W···O2	0.86 (4)	2.24 (5)	2.959 (3)	141 (4)
O1W—H2W···O3^ii^	0.96 (4)	1.91 (4)	2.840 (3)	163 (4)

Symmetry codes:  (ii) *x*, −*y*+1/2, *z*−1/2.
